# Supplementary material for: A developmental atlas of zebrafish gills links early vascular patterning to adult architecture
Source: Development. 2025 Sep 15;152(20):dev204984. doi: 10.1242/dev.204984 (PMC12517346; doi:10.1242/dev.204984)
Supplement: Supplementary information [file develop-152-204984-s1.pdf]

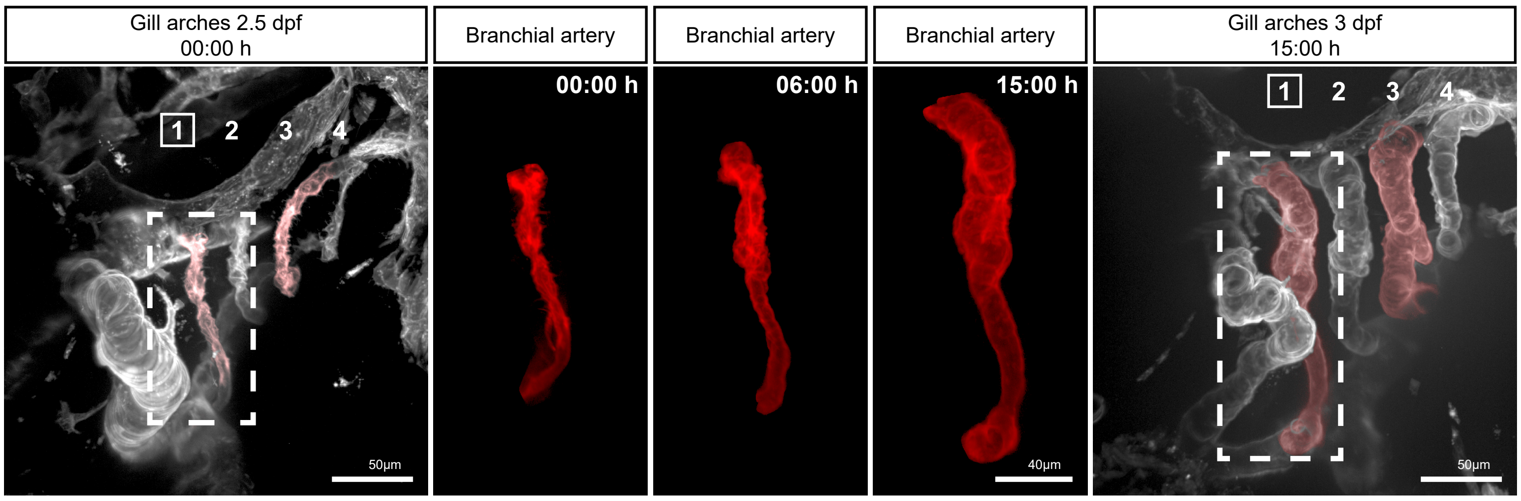

Fig. S1. Branchial arteries expand greatly in embryos transitioning from 2.5 to 3 dpf. Maximum intensity projection (MIP) from a 15-hour timelapse of gill vasculature development in a 2.5 dpf *Tg(kdrl:mCherry)* embryo. The irst arch was digitally isolated at given timepoints to highlight the increase of the arteries diameter.

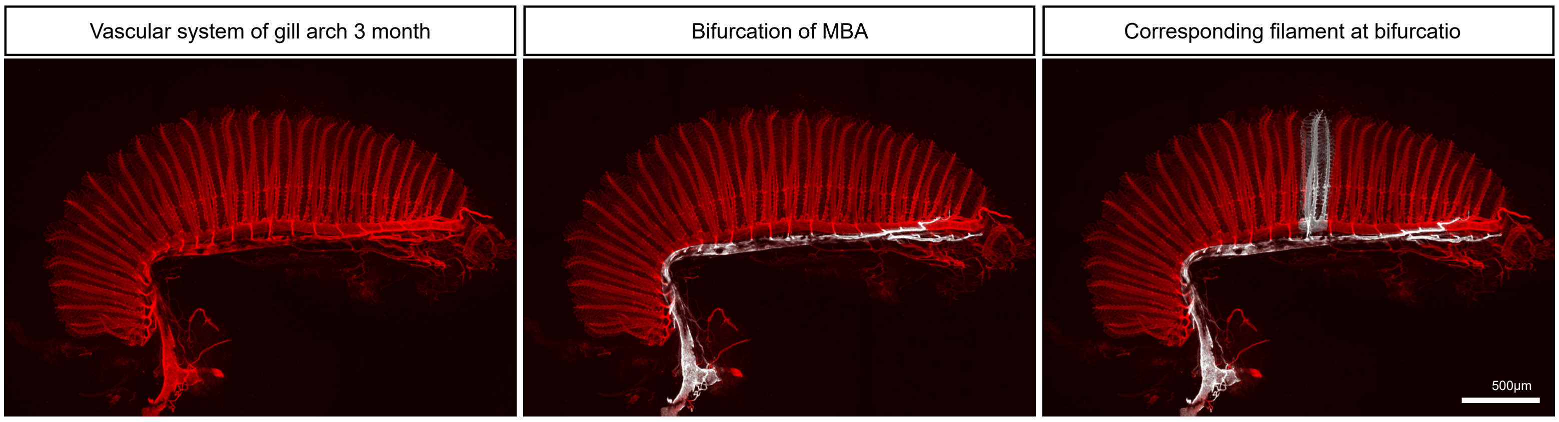

Fig. S2. The MBA bifurcation can still be identified in mature gills. Dissected first gill arch from 3 month-old *Tg(kdrl:mCherry)* zebrafish with pseudo-coloured MBA (middle) and the corresponding filament dorsal to the bifurcation (right) which corresponds to the very first-formed filament.

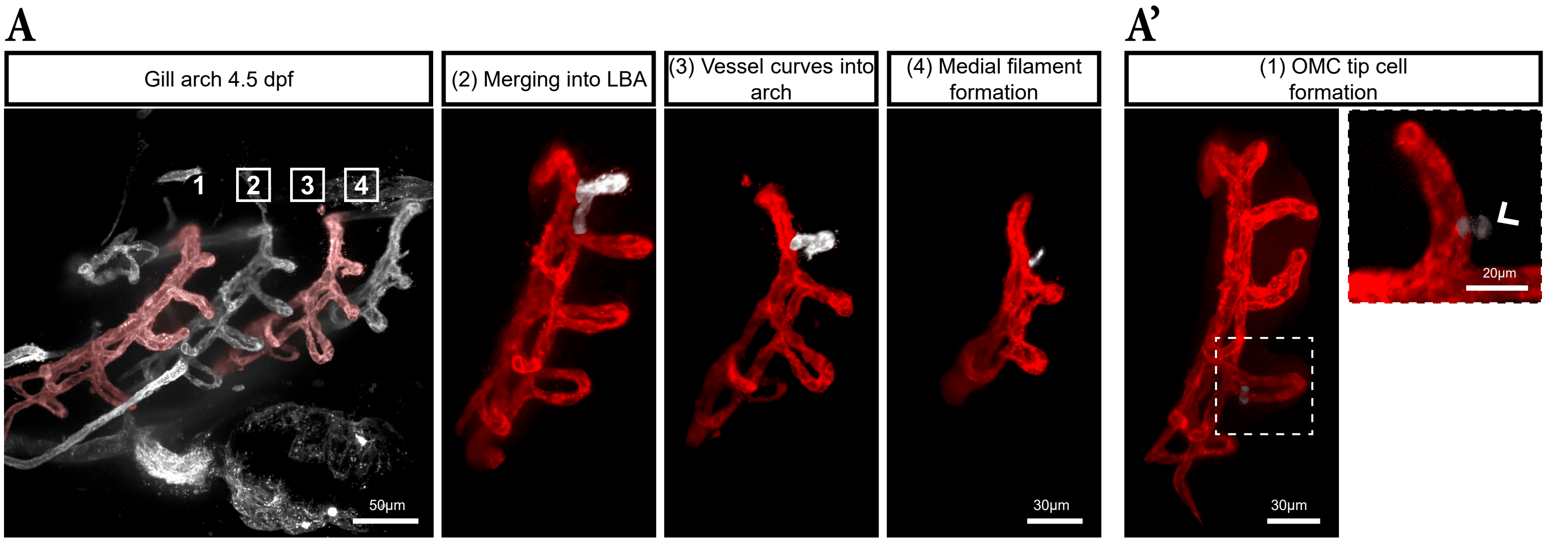

Fig. S3. Dorsal ilament development. (A) Maximum intesity projection (MIP) of the gill arches in fixed 4.5 dpf *Tg(kdrl:mCherry)* embryo. Digital isolated gill arches 4 to 2 highlighting the dorsal development of medial filaments after lateral branchial artery (LBA) formation (pseudo-coloured in white). (A') Digitally isolated first gill arch from overview in A, with close-up view showing the outer marginal channel (OMC) tip cell of an emerging lamella (wide arrowhead on pseudo-col-oured lamella tip cell).

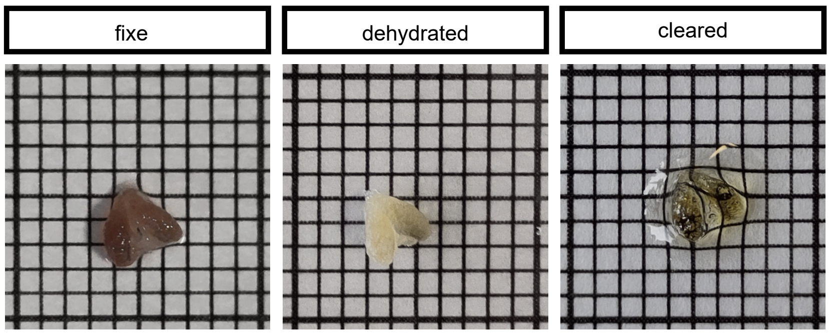

Fig. S4. ECi-based tissue clearing effectively renders gill tissue transparent, enabling high-resolution imaging of internal structures. Millimeter paper provides scaling.

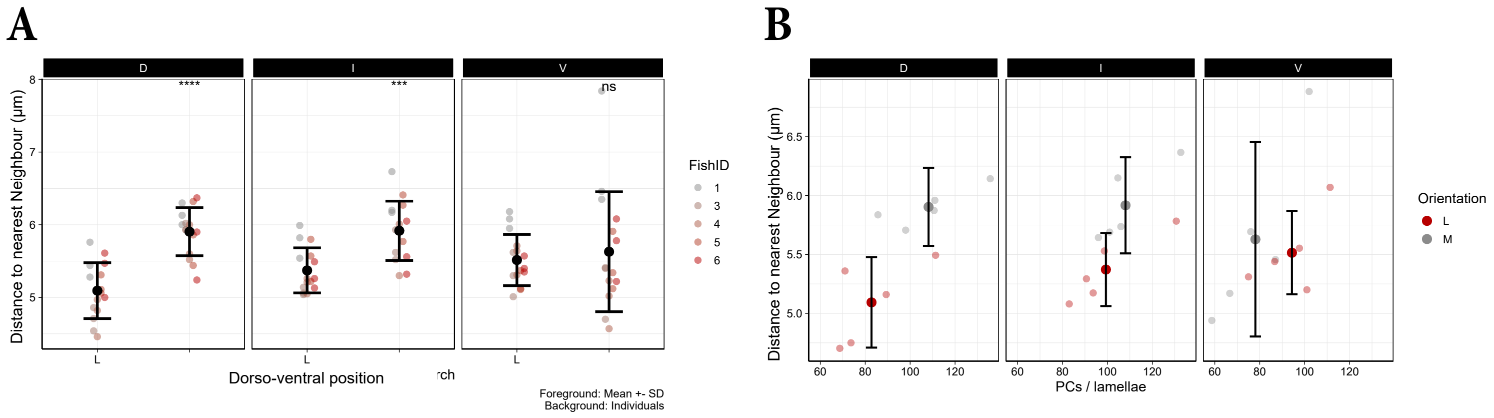

Fig. S5. An increased number of pillar cells within lamellae is associated with greater spacing between them. (A) Comparison of the distance to nearest neighbour (DTNN) of PCs per lamellae in medial (M) versus lateral (L) filaments in the dorsal (D), intermediate (I) and ventral (V) third of the arch. (B) Plot of the distance between PCs as a function of the number of PCs for individual lamellae in the three regions of the arch (D, I and V regions).

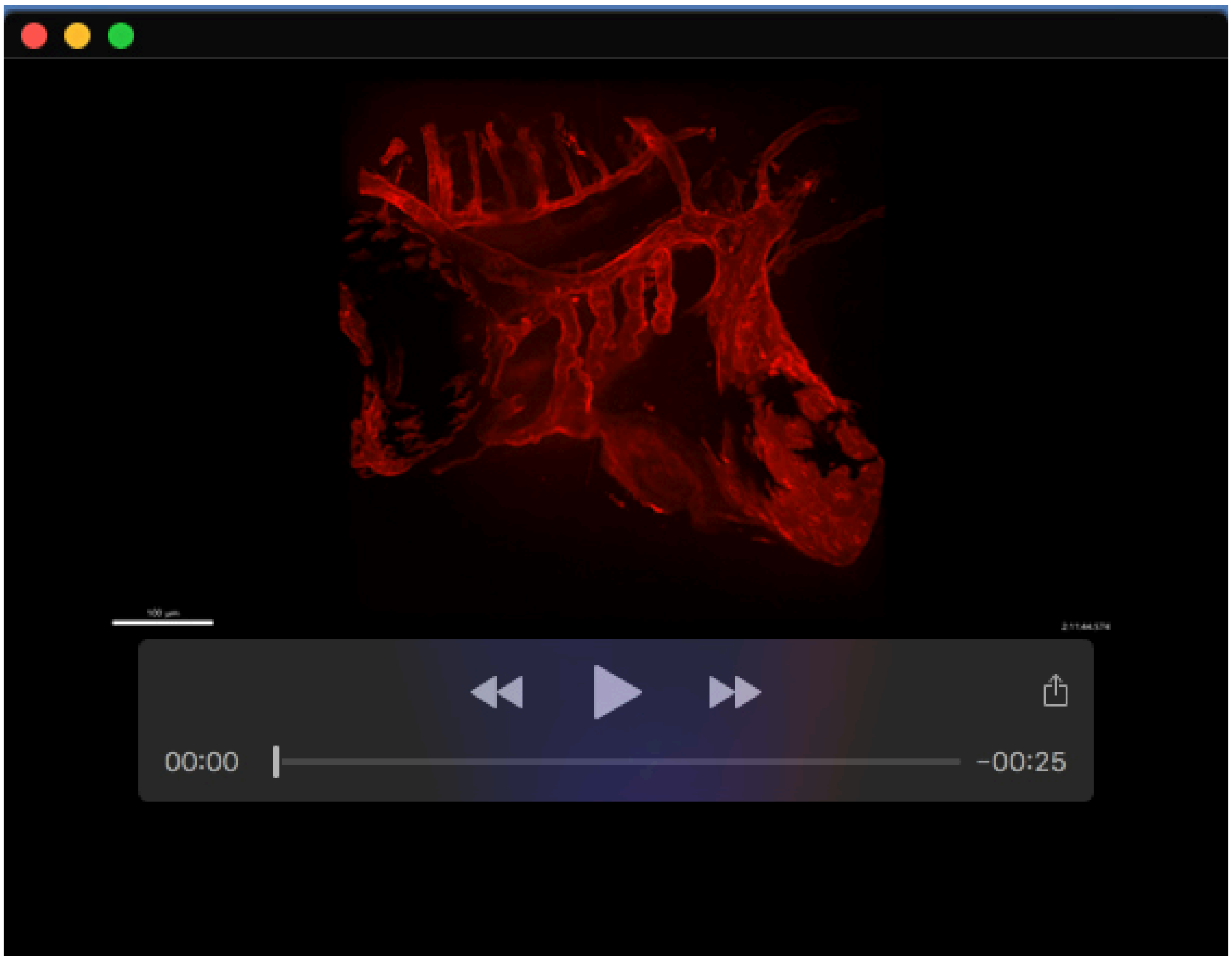

Movie 1. Onset of gill filament formation. 16-hour timelapse of a 3 dpf *Tg(kdrl:mCherry)* embryo showing the onset of gill filament formation (corresponding to Fig. 3A). Digitally zoomed-in frames at selected timepoints highlight the individual steps illustrated in Fig. 3A.

Development • Supplementary information

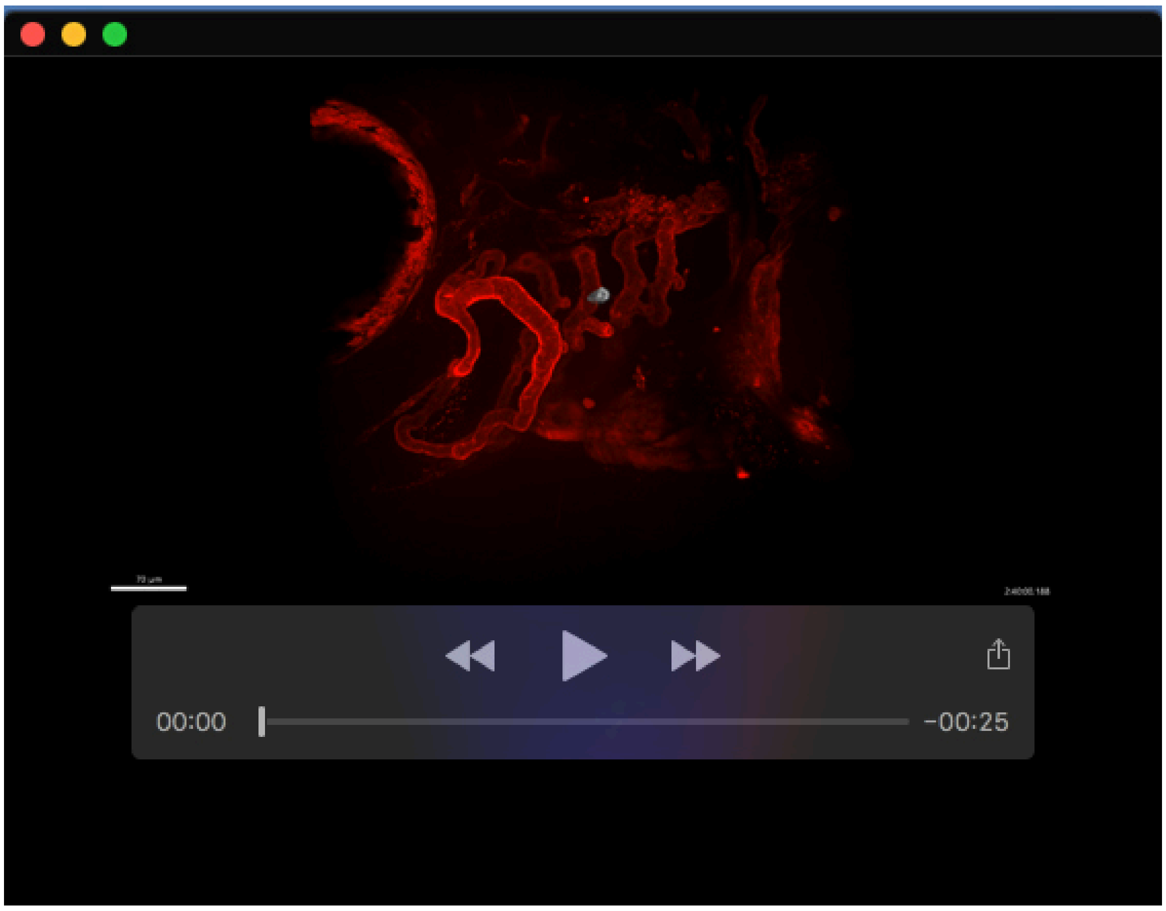

Movie 2. Medial filament formation in the dorsal region of the gill arches. 15-hour timelapse (corresponding to Fig. 4A) of gill vasculature development in a 3.5 dpf *Tg(kdrl:mCherry)* embryo highlighting the formation of medial filaments (pseudo-coloured in white).

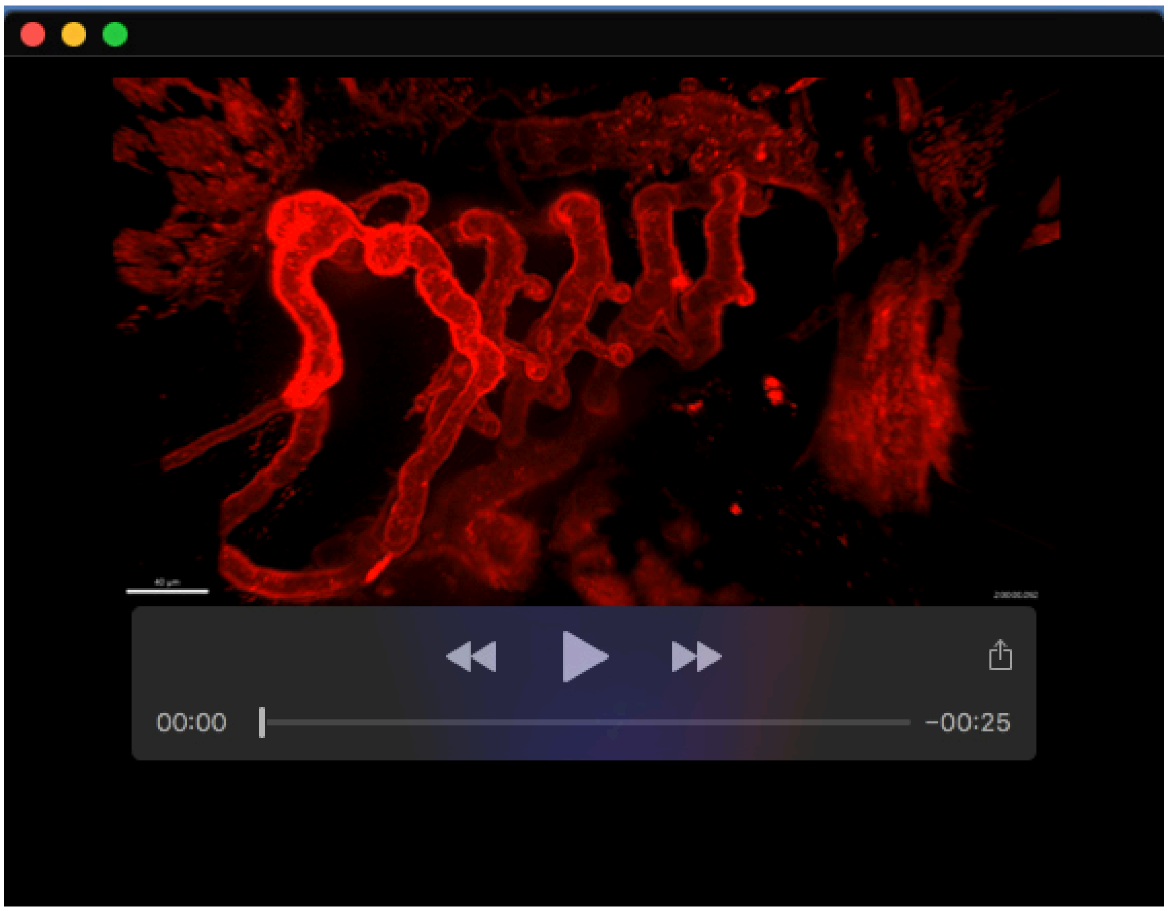

Movie 3. Lateral filament formation in the dorsal region of the gill arches. 15-hour timelapse (corresponding to Fig. 4B) of gill vasculature development in a 3.5 dpf *Tg(kdrl:mCherry)* embryo highlighting the formation of lateral filaments (pseudo-coloured in white).

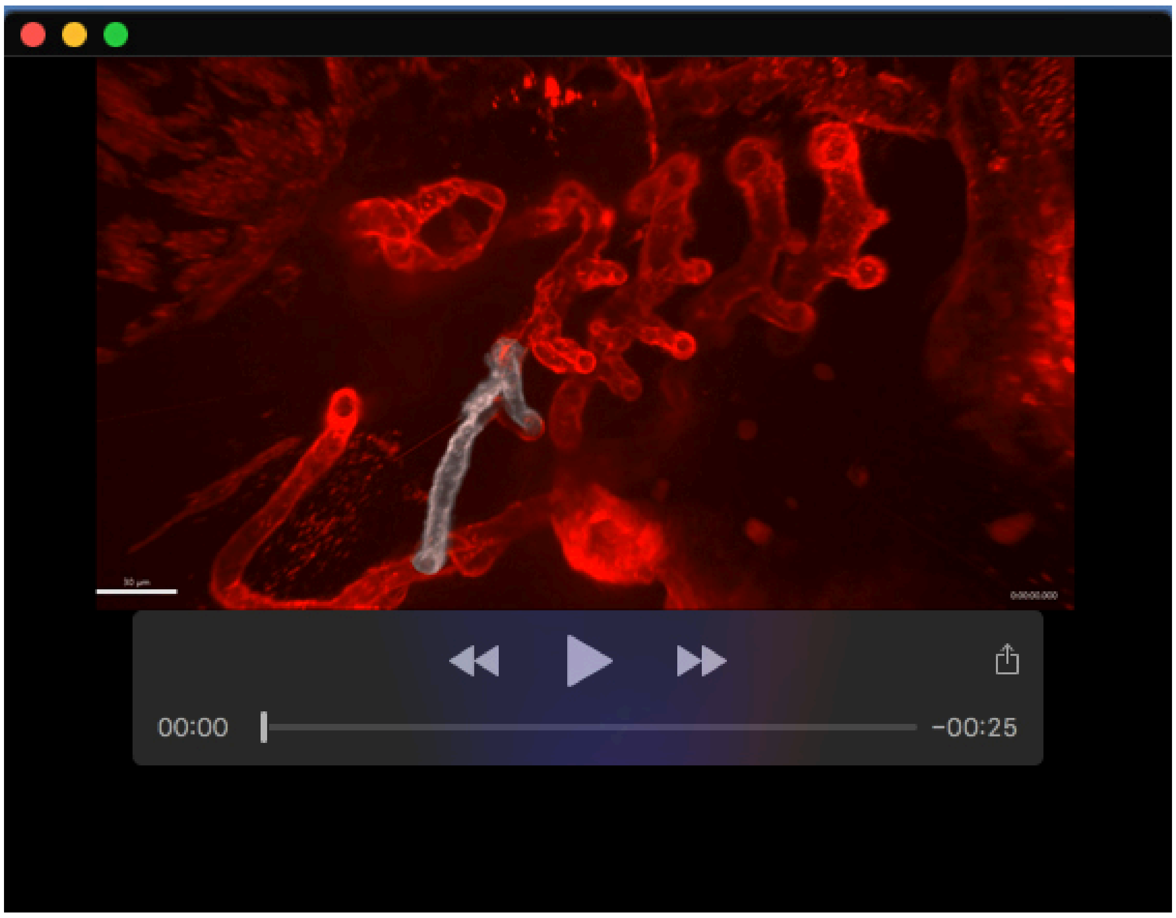

Movie 4. Filament formation in the ventral region of the gill arches. 12-hour timelapse (corresponding to Fig. 5) of the gill vasculature from 4.5 to 5 dpf *Tg(kdrl:mCherry)* embryo. Digitally zoomed-in frames at selected timepoints highlight the individual steps illustrated in Fig. 5B-B', C-C', D-D', E-E'.

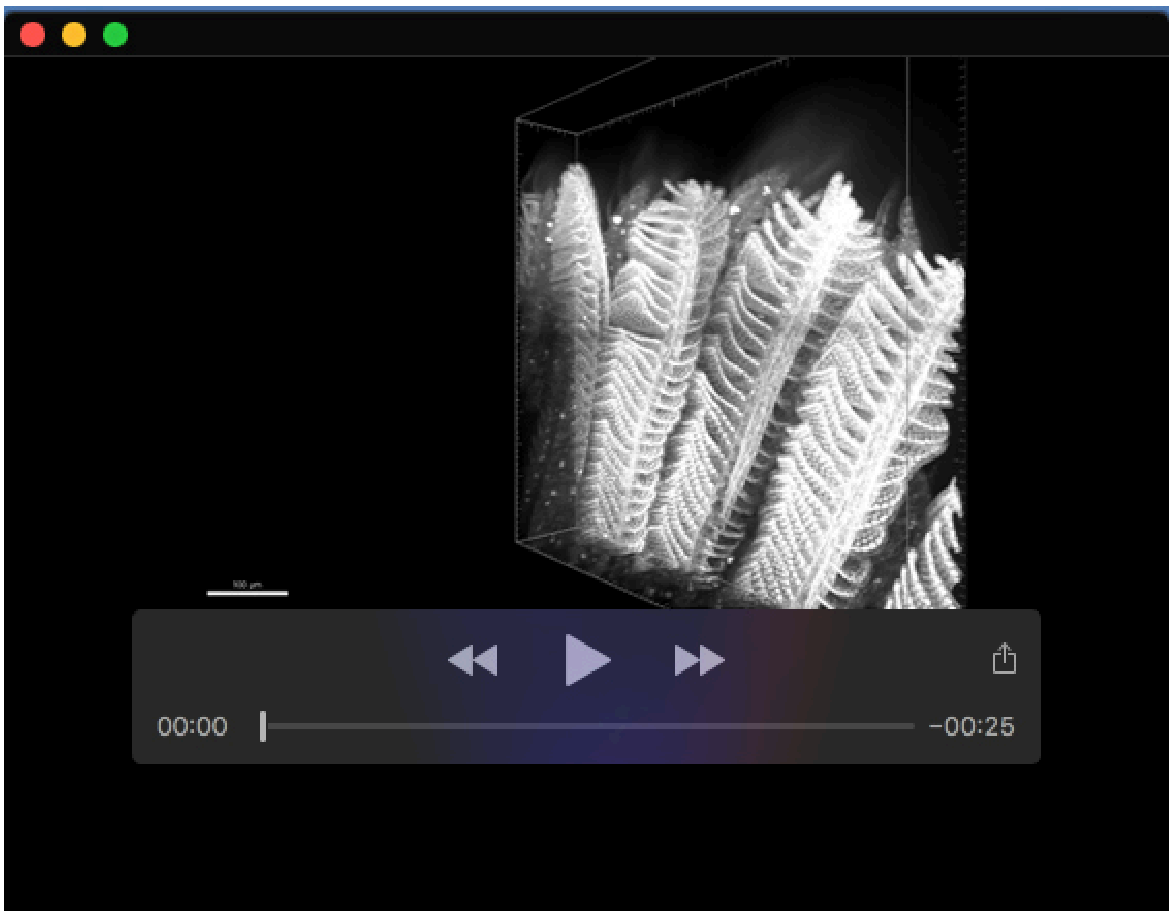

Movie 5. Pipeline of pillar cell quantification in zebrafish gills. PC quantification workflow shown on the same filament and lamellae as in Fig. 10.
